# Deciphering the Relationship between SARS-CoV-2 and Cancer

**DOI:** 10.3390/ijms24097803

**Published:** 2023-04-25

**Authors:** Michele Costanzo, Maria Anna Rachele De Giglio, Giovanni Nicola Roviello

**Affiliations:** 1 Department of Molecular Medicine and Medical Biotechnology, School of Medicine, University of Naples Federico II, Via S. Pansini 5, 80131 Naples, Italy; 2 CEINGE–Biotecnologie Avanzate Franco Salvatore s.c.ar.l., Via G. Salvatore 486, 80145 Naples, Italy; 3 School of Medicine and Surgery, University of Naples Federico II, Via S. Pansini 5, 80131 Naples, Italy; 4 Institute of Biostructures and Bioimaging, Italian National Council for Research (IBB-CNR), Via P. Castellino 111, 80131 Naples, Italy

**Keywords:** oncoviruses, oncogenic virus, oncolytic virus, SARS-CoV-2, long COVID-19, COVIDomics, immunotherapy, immune escape, metabolic reprogramming, coronavirus

## Abstract

Some viruses are known to be associated with the onset of specific cancers. These microorganisms, oncogenic viruses or oncoviruses, can convert normal cells into cancer cells by modulating the central metabolic pathways or hampering genomic integrity mechanisms, consequently inhibiting the apoptotic machinery and/or enhancing cell proliferation. Seven oncogenic viruses are known to promote tumorigenesis in humans: human papillomavirus (HPV), hepatitis B and C viruses (HBV, HCV), Epstein-Barr virus (EBV), human T-cell leukemia virus 1 (HTLV-1), Kaposi sarcoma-associated herpesvirus (KSHV), and Merkel cell polyomavirus (MCPyV). Recent research indicates that SARS-CoV-2 infection and COVID-19 progression may predispose recovered patients to cancer onset and accelerate cancer development. This hypothesis is based on the growing evidence regarding the ability of SARS-CoV-2 to modulate oncogenic pathways, promoting chronic low-grade inflammation and causing tissue damage. Herein, we summarize the main relationships known to date between virus infection and cancer, providing a summary of the proposed biochemical mechanisms behind the cellular transformation. Mechanistically, DNA viruses (such as HPV, HBV, EBV, and MCPyV) encode their virus oncogenes. In contrast, RNA viruses (like HCV, HTLV-1) may encode oncogenes or trigger host oncogenes through cis-/-trans activation leading to different types of cancer. As for SARS-CoV-2, its role as an oncogenic virus seems to occur through the inhibition of oncosuppressors or controlling the metabolic and autophagy pathways in the infected cells. However, these effects could be significant in particular scenarios like those linked to severe COVID-19 or long COVID. On the other hand, looking at the SARS-CoV-2─cancer relationship from an opposite perspective, oncolytic effects and anti-tumor immune response were triggered by SARS-CoV-2 infection in some cases. In summary, our work aims to recall comprehensive attention from the scientific community to elucidate the effects of SARS-CoV-2 and, more in general, β-coronavirus infection on cancer susceptibility for cancer prevention or supporting therapeutic approaches.

## 1. Introduction

Cancer is still a global threat that seriously affects human life, with a prevalence higher than 10 million deaths yearly [1]. Despite the successful efforts in increasing cancer-free survival rates, many cancer therapies lead to severe undesirable side effects, thus limiting the therapeutic options for cancer treatment [2].

The early diagnosis of cancer and the correct diagnosis, followed by an accurate characterization of the cancer type, are crucial steps in managing cancer patients to increase their survival probability, being the late diagnosis after emergency presentation associated with poor prognosis [3,4,5].

In this context, the already-disposable therapies may be effective only on a restricted number of cancers. Furthermore, the (single or cumulative) events that increase the mutation rate of genes involved in cellular proliferation, DNA repair, or apoptosis correlate with cancer incidence. Among the cancer-inducing events, those triggered by viruses, hence called oncogenic viruses or oncoviruses, are remarkable for being significantly fatal [6,7,8], and their therapy can scarcely improve life expectations in patients. Some virus strains are highly pathogenic *per se*, and the early diagnosis or the antiviral therapies are often not adequately contemplated. This is often recognized when some oncogenic viruses, coevolving with their asymptomatic human hosts, manifest latent or chronic infections [9]. These viruses may become part of the microbial community of the human host together with other viruses, constituting the so-called human virome [10]. In particular cases, if not pathogenic, they may positively contribute to human health [11]. Virus infections have recognized causal roles in developing several tumors in humans or animals, accounting for around one-fifth of the total cancers [12]. In particular, the oncogenic viruses are estimated to be connected with around 15–20% of all human cancers, providing each individual with a ‘risk factor’ of generating tumors caused by virus infection [13,14,15,16].

The relationships that interconnect viruses and cancer have represented one of the most outstanding discoveries in virology and oncology [6]. Owing to their ability to predispose to tumor development, the oncogenic potential of viruses easily drew the attention of the scientific community. Being initially controversial, the bases of human viral oncogenesis have been extensively explored, establishing common traits and thus defining the viral cancer hallmarks [8]. These include the following considerations: (i) oncogenic viruses are indispensable but not self-sufficient for the genesis of tumors; in the human population the incidence of cancer is significantly inferior with respect to the prevalence of the oncoviruses; (ii) virus-induced cancers emerge in combination with lasting infection; they can appear even several years after the acute infection; (iii) the immune system largely modulates the development of viral cancers. The latter might inhibit the tumor growth or promote it in the context of immunosuppression or chronic inflammation [8]. However, the major challenge of viral oncology remains to determine and characterize the exact pathways and mechanisms behind the processes of viral oncogenesis, which depends on many viral oncogenes and factors able to favor cellular transformation. While few mechanisms can be finely characterized even in particular tumors or tissues, others result more intricate, thus limiting the association of cancer with a particular causal agent or a single cellular event. Contrarily, many oncogenic mechanisms have been recognized to act on diverse spots of the host cell signaling machinery [17,18,19]. For example, some insights reveal novel interconnections between autophagy and mitochondrial metabolism in cancer cells [20]. This evidence highlights the need to dissect the molecular effects of the single oncoviral event and understand how multiple or cumulating oncogenic events can modulate cancer onset and progression [14]. Pioneering studies at the beginning of the twentieth century have identified several oncoviruses potentially able to induce tumoral transformation in animals [21,22]. Later, these oncogenic abilities were recognized even in human viruses [23,24].

Conversely, most of the viral oncogenes were then identified as genes of the host organism acquired through viral recombination. These oncogenes showed their oncogenic potential in the virus-infected cells by accumulating gain-of-function mutations with the subsequent alteration of gene expression; or expressing molecules able to functionally inhibit the p53 and Rb oncosuppressors [25]. It is currently acknowledged that the classical viral oncogenes are insufficient for cancer development [14], despite their potential to induce tumoral transformation in experimental models. Other viruses may generate human cancer only in particular situations, with most virus-induced tumors remaining steady as benign infections [26]. It is acknowledged that the biochemical pathways perturbed by viral disease and their association with human cancers hold a certain molecular intricacy.

Nonetheless, according to the growing interest in virus-induced pathologies, a controversial role for the severe acute respiratory syndrome coronavirus 2 (SARS-CoV-2) in holding an oncogenic potential is debated as advertised by recent literature. Hence, SARS-CoV-2 may favor tumor progression by acting on metabolic reprogramming to stimulate cell replication. On the other hand, an anti-tumor immune response was observed in some lymphoma patients, providing evidence for a potential oncolytic role for this coronavirus [27]. For these reasons, the mechanisms by which SARS-CoV-2 can modulate cellular oncogenic-related pathways still need to be explored.

## 2. Oncogenic Viruses and Their Mechanisms

Mainly, seven oncoviruses are known to promote the process of tumorigenesis, namely the human papillomavirus (HPV) [28,29,30,31], the hepatitis B and C viruses (HBV, HCV) [32,33,34,35,36], the Epstein-Barr virus (EBV) [37], the human T-cell leukemia virus 1 (HTLV-1) [38,39], the Kaposi sarcoma-associated herpesvirus (KSHV), also known as human herpesvirus-8 (HHV-8) [40,41], and the Merkel cell polyomavirus (MCPyV) [42,43]. Other potential cases of tumor-inducing viruses may be represented by the human cytomegalovirus (CMV), whose tumorigenic potential is still under investigation [44], as well as the human herpesvirus-6 and the adeno-associated virus-2 [11]. Finally, the human immunodeficiency virus 1 (HIV-1) is indirectly connected with a certain risk of developing cancer upon its mechanisms of immunosuppression [11]. While a comprehensive review based on the oncogenic viruses is out of the scope of this article, here we briefly discuss the seven oncoviruses mainly involved in the carcinogenesis, recapitulating their proposed mechanisms of action, and extracting some elements useful in the context of the SARS-CoV-2 infection.

All the exact biochemical pathways perturbed by virus infections remain undiscovered. The small-size genomes of these viruses range from a few Kb to around 200 Kb, not ensuring an extensive coding ability. As an adaptive consequence, the oncoviruses depend on the host cell proteome to hijack the proliferation of cellular pathways, thus harnessing the whole cell [45]. Therefore, the oncogenesis endorses a multi-step process to be promoted, from the in situ formation of the tumor to the generation of circulating metastatic cells, which depends on the differential regulation of proliferation, apoptosis, and senescence pathways [45]. The seven oncoviruses functionally dysregulate the host cellular pathways involved in cell cycle progression and apoptosis to sustain their propagation. However, despite being jointly characterized by similar mechanisms of pathological infection, the oncogenesis is not indispensable for virus spread from an evolutionary point of view [46]. Some data on the global burden sustained by viral cancers, according to the data collected by a recent review [46], are reported in Table 1.

DNA viruses (HPV, HBV, EBV, HHV-8, MCPyV) encode their virus oncogenes, while RNA viruses (HCV, HTLV-1) may encode oncogenes or trigger host oncogenes through cis-/-trans activation. Oncoviruses may act using different oncogenic mechanisms classified as direct or indirect [47]. In general, direct oncogenesis implicates the insertion of viral oncogenes into the host cell or can be promoted by activating oncogenes already existing in the genome (proto-oncogenes). Indirect viral oncogenesis is promoted by chronic non-specific inflammation occurring over decades of infection, possibly after virus latency inactivity, as for the hepatic cancers induced by HCV. In addition, viruses can integrate their DNA sequences with oncogenic roles. For example, RNA viruses can reverse-transcribe their genome into double-stranded DNA sequences (proviruses) that become successively integrated into the host genome [27]. Specifically, oncogene-containing retroviruses may insert their sequences to enable the transcription of the genes.

On the other hand, oncogene-lacking retroviruses might constitutively activate host proto-oncogenes through proviruses insertion in the nearby proto-oncogene regulatory sequences (insertional mutagenesis); viral promotors take control of the host proto-oncogenes mediating their constitutive activation [27,48] In fact, viral integration into the host genome has been revealed to be a causal mechanism leading to tumor development [49,50], as the additional insertional mutagenesis favors the generation rate of oncogenic mutations, concurring to the genomic instability. Meanly, oncoviruses may straightforwardly trigger the host cell transformation through (i) the integration of a viral oncogene (or only a part of its sequence) into the cellular genome, (ii) the overactivation of human oncogenes, or (iii) the inhibition of tumor suppressors [8]. Thus, the regulation between cell cycle and death signaling is averagely compromised as a target mechanism common to all the oncogenic viruses, despite the fact they express diverse viral products. Moreover, oncoviruses inactivate tumor suppressors and potentiate oncogenes transcription, thus modulating the expression and function of several protein actors and related signaling pathways besides the renowned p53 and pRB, TNF, MAPK, PI3K-AKT-mTOR, WNT/β-catenin, NF-κB and interferon signaling pathways [46,51,52].

**Figure 1:**
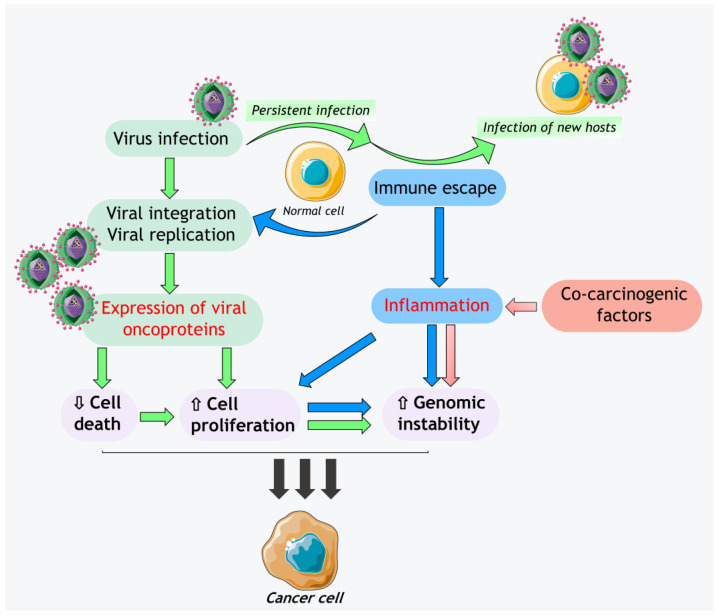
Schematic representation of the cross-talks between viral (green boxes) and host (blue boxes) events contributing to oncogenesis in human cells. Human oncoviruses infect the cells by establishing persistent infections that reprogram the host metabolism, subverting through the immune escaping the antiviral defense of the cell. When the virus can replicate massively, it can infect new hosts. An oncovirus free of replicating inside the cell actives the expression of viral oncoproteins or the transcription of host genes that mediate the cell survival through increased proliferation and reduced cell death. With the increased rate of cell cycles, the likelihood of events that cause genomic instability is also increased. Genomic instability is also promoted by the inflammation deriving from the escape of the immune system and external co-carcinogenic factors (red box). The inflammatory response to oncovirus infection generates ROS that augments the genomic instability, also through the potentiation of cell proliferation to replace damaged tissues. The combination of all these factors (purple boxes) can contribute to the transformation of a normal cell into a cancer cell.

Established that cancer development arises from uncontrolled proliferation stimuli and cellular immortality, this complex and multidimensional scenario is accompanied by multiple metabolic dysregulations, immune response escaping, induction of inflammation with the production of reactive oxygen species (ROS), generation of a proper tumor microenvironment, and the genomic instability itself [53,54]. Furthermore, genomic instability and phenotype are further targeted by the generation of genetic and epigenetic changes during the numerous replication cycles, such as DNA methylation and histone modifications, or worsened by co-carcinogenic factors and external stimuli [55,56] (Figure 1).

## 3. The Oncogenic Potential of SARS-CoV-2

The coronavirus disease 19 (COVID-19) caused by SARS-CoV-2 was responsible for huge sanitary and socio-economic difficulties experienced all across the entire world because of the high transmission rates of the virus, its pathogenicity and the lack of effective COVID-19 treatments [57,58,59,60,61] or vaccines [62,63,64] available when it first emerged, and the rapid genetic mutational conversion observed in the last years [65,66,67].

The effects of SARS-CoV-2 infection on cancer patients have been largely investigated for their care and management. It was observed that patients with solid cancer or a hematologic malignancy were more prone to be infected, showing increased morbidity and mortality when compared to the rest of the population [68]. In addition, compared with other tumor types, patients with hematological cancer were more prone to mortality events considering that the dysfunctional immune cells linked to hematopoietic malignancies can significantly shut down the immune defenses of an individual [69].

Several reports have explained that the metabolic perturbance deriving from SARS-CoV-2 infection is the cause of the systemic alterations persisting in COVID-19 patients, especially those characterized by severe symptoms. In this scenario, metabolic reprogramming has been considered a distinctive feature of SARS-CoV-2 [70,71,72,73]. This event is caused by the replication of SARS-CoV-2 for its survival and is modulated by the host immunity activated by the viral infection [71,74]. Several metabolic pathways and processes have been investigated by omics technologies and discovered as reprogrammed in hospitalized COVID-19 patients, including amino acid and lipid metabolism, carbohydrate and energy metabolism, and immune-related pathways [75,76,77,78,79].

With such evidence, it becomes clear that the modifications induced by SARS-CoV-2 are substantial for its survival in the host. Additionally, some major signaling pathways have been recognized at the cross-talk between SARS-CoV-2 and cancer cells, frequently stimulating the tumor progression or modifying the response of the tumor to therapy. However, the causal relationship between SARS-CoV-2 and cancer and the effective role of the virus in oncogenesis still represents an open question, considering the observed reactivation of oncogenic viruses following COVID-19 in some cases and the paradoxical response of certain tumors to the immune modulation induced by the infection in others [80].

Similar to oncoviruses, SARS-CoV-2 would be able to promote cancer progression through the alteration of central metabolic pathways in tumor cells and in patients, such as carbon and nitrogen metabolism and nucleic acid metabolism [75,81]. It was found that human biofluids, as well as the infection of Caco-2 (human colon epithelial carcinoma) cells by SARS-CoV-2, affected the proteome negatively regulating the expression of cholesterol-related proteins and positively regulating carbohydrate metabolism-related proteins [27,75,81]. Accordingly, SARS-CoV-2 could excite a metabolic switch in tumor cells to support high-energy production pathways, i.e., glycolysis, for sustaining its replication rate [82,83].

Despite the controversial debate about the oncogenic (or oncolytic) potential of SARS-CoV-2, several genes with a role in oncogenesis have been found regulated upon its infection, such as those corresponding to E2F transcription factors and pRB, thus suggesting a putative mechanism for SARS-CoV-2 in contributing to oncogenesis through the potential inhibition of oncosuppressors [84]. Interactomics studies were pivotal to obtaining such mechanistic insights [85]. Particularly, it was described that the interaction between the endoribonuclease non-structural protein 15 (Nsp15) of SARS-CoV-2 and pRB induces the nuclear export and ubiquitination of pRB for its degradation via proteasome [86]. Furthermore, NIH-3T cells that express the Nsp15 protein did not preserve contact inhibition, displaying an amplified proliferative potential for the induction of cellular transformation [86].

**Figure 2:**
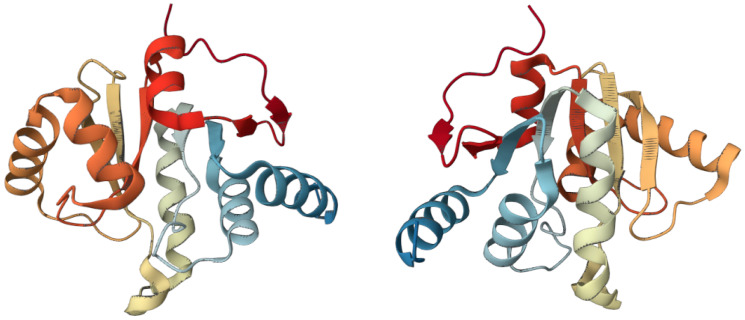
Two 3D views of Nsp3 macrodomain of SARS-CoV-2. (PDB ID: 6WEY; https://www.rcsb.org/3d-view/6WEY/1, link accessed on 23 February 2023) [90].

A second potential oncogenic mechanism has been hypothesized for SARS-CoV-2 consisting of the degradation of p53 mediated by the non-structural protein 3 (Nsp3). As previously shown for SARS-CoV-1, the papain-like protease (PL^pro^) domain of Nsp3 interacts with and stabilizes the E3 ubiquitin ligase RCHY1 [87], thereby promoting the RCHY1-mediated degradation of p53 [88,89]. Furthermore, the Nsp3 protein is highly conserved between SARS-CoV-1 and SARS-CoV-2, showing 76% of sequence similarity. This similarity strongly suggests that SARS-CoV-2 Nsp3 (Figure 2) may drive the potential to lower p53 levels promoting its degradation, thus increasing the probability of cellular transformation [6].

The SARS-CoV-2 could provide additional mechanisms to control p53 degradation by hijacking the protein through viral antigens [91,92]. Precisely, the Nsp2 protein of the SARS-CoV-2 interacts with the prohibitins 1 and 2 (PHB1, PHB2) that function as chaperones in the inner mitochondrial membrane for stabilizing the mitochondrial respiratory enzymes and maintaining the mitochondrial integrity. Furthermore, their depletion activates a cascade of cellular responses that prime the leakage of ROS to the nucleus with subsequent oxidative damage, finally impairing the transactivation of p53-dependent genes [92]. Although not demonstrated yet, the ability of the proteins of SARS-CoV-2 to inhibit both p53 and pRB by mediating their degradation suggests that SARS-CoV-2 may have oncogenic potential, triggering internal and external apoptotic pathways within the host cell.

Cancer progression may be potentially favored by SARS-CoV-2-mediated modulation of macro-autophagy/autophagy, proved that diverse coronaviruses can regulate the autophagic machinery [93]. A particular form of autophagy by which the endoplasmic reticulum (ER) is selectively degraded (ER-phagy) seems to be modulated by coronaviruses to drive the formation of double-membrane vesicles (DMVs) that serve as viral replication organelles. Precisely, it was demonstrated that the open reading frame 8 (ORF8) protein of SARS-CoV-2 interacts with p62, the main autophagic cargo receptor, showing that the ORF8/p62 complexes hamper ER-phagy by inhibiting the ER-phagy receptors FAM134B and ATL3 through their aggregation into ORF8/p62 liquid droplets. This mechanism disrupts ER-phagy to promote the formation of new viral DMVs and activation of the ER stress [94]. In addition, it was reported that ORF8 protein directly interacts with major histocompatibility complex class I (MHC-I) molecules, mediating their down-regulation. In particular, SARS-CoV-2-infected cells were significantly less susceptible to cytotoxic T lymphocyte-mediated lysis, being MHC-I molecules selectively targeted for lysosomal degradation via autophagy. Thus, SARS-CoV-2 infection could arbitrate immune evasion through down-regulating MHC-I and impairing the antigen presentation system [95].

The role of autophagy in cancer has a miscellaneous facet, with several activities that facilitate cancer cells proliferation and survival, as well as migration and invasion, through recycling metabolites for their growth, regulating their mitochondrial tasks via mitophagy, or controlling the turnover of cell adhesion and the secretion of pro-migratory and inflammatory cytokines, along with adaptation to the microenvironment [96,97]. Modulation of autophagy supports the proliferation of cancerous cells and their survival. Hence, with the ability of SARS-CoV-2 to control to a certain extent the degradation pathways in the cells, cancerogenesis may be promoted by the viral-mediated subversion of autophagy machinery and organelle-specific autophagy.

Further evaluation of a possible correlation between SARS-CoV-2 and cancer arises from the findings of elevated mucin (MUC) levels during COVID-19 infection in patients. MUC glycoproteins are the major macromolecular components of mucus, essential in maintaining the function of districts such as the lung and intestine. In particular, MUC1 is a membrane-bound mucin that shows high expression in the apical membranes of the bronchial epithelium and the gastrointestinal tract. MUC5AC is a secretory mucin expressed mainly in the gastric and tracheobronchial lining. In some cancer-related conditions, glycosylated MUC is abnormally overexpressed by tumors and secreted in the circulation of patients, serving as tumoral biomarkers. Increased MUC1 and MUC5AC mucin protein levels were found in the airway mucus of critically ill COVID-19 patients [98]. In addition, the carbohydrate antigen 72-4 (CA 72-4) marker increased during COVID-19 infection in patients [99]. CA72-4 is a type of cancer-associated polymorphic epithelial MUC, highly expressed in human adenocarcinomas, including gastric, colon, breast, and lung cancer, showing low levels in normal tissues instead. CA72-4 is especially used as an indicator for the tumors of the digestive system [100]. These findings do not provide evidence for direct cancer development but certainly show a possible connection with the onset of tumors in infected patients.

Finally, an alarming situation characterizes COVID-19 patients that do not recover in little time but show sequelae of SARS-CoV-2 infections lasting for months, a condition named as long COVID-19. It has been proposed that long COVID-19 can predispose recovered patients to develop cancer and accelerate cancer progression. This hypothesis has been structured on the mounting evidence of the ability of SARS-CoV-2 to regulate oncogenic pathways, promoting chronic low-grade inflammation and causing tissue damage [101]. Thus, the effects of long COVID-19 on cancer susceptibility need a more profound investigation. In contrast, long-term inhibition of p53 and pRB could be interpreted as an essential risk factor for carcinogenesis.

Long-term relationships between viruses and their hosts are needed for cancer transformation, development, and growth. This is the main reason for the arguments against accurately classifying SARS-CoV-2 as an oncogenic virus. In contrast with classical oncoviruses, and despite the SARS-CoV-2 may exert in vitro oncogenic effects, most infections are resolved in a limited time. Therefore, stating that SARS-CoV-2 is not likely to maintain extremely long-lasting infections opposes its putative role in cancer onset.

## 4. The Oncolytic Potential of SARS-CoV-2

Oncolytic viruses represent a group of viruses that can kill cancer cells, so they are employed as anti-cancer immunotherapy [102,103,104,105,106]. Being able to massively replicate in cancer cells, oncolytic viruses mediate their cell death through a lytic mechanism [107,108]. Currently, the clinical management of immune-treated patients recommends a combination of oncolytic viruses with PD-1/PD-L1 antibodies. In contrast, the block of the PD-1/PDL-1 pathway permits the inhibition of acute or chronic viral infections. This treatment combination is especially effective in patients with malignancies resistant to PD-1/PD-L1 blockade therapy, boosting the anti-tumor immune response [109,110]. In addition, the antiviral response of the immune system triggered by oncolytic viruses can raise the levels of interferons in the cancer environment, thus stimulating the synthesis of PD-L1 for immune evasion [27,110].

The oncolytic potential and the anti-tumor immune response elicited by SARS-CoV-2 have been associated with patients with NK/T-cell lymphoma [27,111]. The massive expression on natural killer (NK) cells of ACE2, which is identified as the main target for the entry of SARS-CoV-2 into host cells [112], makes them easily infectable by the coronavirus, finally provoking a loss of the immune surveillance due to a decline in immune cells number [113]. In particular, an interesting case of transient remission was reported for a refractory NK/T-cell lymphoma during SARS-CoV-2 infection, with its relapse after COVID-19 resolution [111]. Notably, the viral load of EBV-DNA, which is used as a biomarker of NK/T cell lymphoma, dropped during acute SARS-CoV-2 infection, and was recovered as COVID-19 was resolved. It was hypothesized that the massive production of proinflammatory cytokines during COVID-19 could lead to lymph node clearance, as IL-6 and IL-10 markedly reduced the cytotoxicity of NK cells, and IL-2 and TNF-α recruited NK and T cells into the tumor tissue [27,111]. Thus, the depletion and inactivation of NK cells may play an important role in treating patients with NK lymphoma. These observations account for an oncolytic effect of SARS-CoV-2 for lymphoma patients, showing an anti-tumor outcome to a certain extent.

Additionally, recent investigations have proposed a protective role for SARS-CoV-2 infection against Hodgkin lymphoma, favoring an anti-tumoral immune response [114]. This was the case of a 61-year-old man diagnosed with an EBV-positive classical Hodgkin lymphoma concurrently positive for SARS-CoV-2 infection. The patient, discharged after a few days with no corticosteroid or immunochemotherapy treatments, showed, after four months of COVID-19 recovery, reduced lymphadenopathy, and decreased levels of tumor-related biomarkers, as well as EBV viral copies. Therefore, it was hypothesized that an anti-tumor immune response had been triggered by SARS-CoV-2 infection, possibly resulting from the combined effects of cross-reactivity of pathogen-specific T cells with tumoral antigens and the activation of NK cells prompted by the proinflammatory cytokine storm produced in response to COVID-19 [114].

Hence, combining the oncolytic characteristics of SARS-CoV-2 with the memory of T cells through genetic modification could help recall the immunity of known antigens to coronaviruses [27]. As a lesson learnt from SARS-CoV-2, this scenario may serve as the basis for developing novel, potentially effective therapeutic anti-tumor strategies based on oncolytic viruses. However, the effects of the coronavirus as an oncolytic virus seem transient and non-specific and need further investigation.

## 5. Conclusions

Despite the possibilities offered in the post-genomic era to meticulously dissect central and peripherical biochemical pathways [115,116], the causal relationships between oncoviruses and the malignant transformation of human cells are particularly intricate and still need detailed assessment. For example, viruses can hijack the host cellular metabolism for their survival and replication, whereas the metabolic reprogramming induced by oncoviruses would be crucial for malignant transformation. Remarkably, the analysis of the metabolic profiles in virus-induced cancers may offer the possibility of discovering new targets for tumor prevention and treatment and developing diagnostic and therapeutic strategies such as the targeted synthesis of molecules for potential anti-oxidant approaches [117,118]. However, metabolic profiling may be paramount in discovering critical information to drive tailored treatments in a contest of precision medicine, even in case of scarce enrollment of patients or in the presence of confounding factors for biomarkers discovery and prevention approaches [119,120,121,122].

Notably, compared with the classical oncoviruses and their mechanistic behaviors in inducing the onset of cancer, some differences with SARS-CoV-2 have been contemplated. In particular, while DNA viruses (such as HPV, HBV, EBV, and MCPyV) encode virus oncogenes, and RNA viruses (like HCV, HTLV-1) encode oncogenes or trigger host oncogenes through cis-/-trans activation, the role of SARS-CoV-2 as oncovirus seems to occur through the inhibition of oncosuppressors as testified by a growing body of evidence that allows speculating about this hypothesis, even though some studies have led to controversial conclusions. The oncogenic potential for the COVID-19-causing β-coronavirus could be explicated by inhibiting the tumor suppressors pRB and p53 through the activities and interactions of the SARS-CoV-2 proteome. While the virus could promote cell transformation by promoting the oncogenic pathways, in concert with the generation of inflammation, tissue damage events and immune escape, on the other hand, the transient nature of the infection and its rapid resolution argue against the likelihood of transformation into cancer cells. Different evidences suggest that the most significant contribution of SARS-CoV-2 to tumor onset may be indirect and related to the extensive lung fibrosis that characterizes severe COVID-19 [6,123], whereas the long-term effects prompted by long COVID-19 are still obscure. In conclusion, with the jury still out on the role of SARS-CoV-2 as an oncovirus, most articles report significant action of SARS-CoV-2 on having an oncogenic potential. Looking at the situation from a relative perspective, the evidence certainly moves the needle in favor of a viral oncogenic potential rather than an oncolytic one.

## Figures and Tables

**Table 1:** The seven oncoviruses associated with the prevalent cancers caused and the world’s major regions affected.

Virus	Cancer	Main Geographic Area
Human papillomavirus (HPV)	>95% of cervical carcinoma 70% of oropharyngeal carcinoma Other anogenital carcinomas	Asia Central America South America Sub-Saharan Africa
Hepatitis B virus (HBV)	53% of hepatocellular carcinoma	Asia South America Sub-Saharan Africa
Hepatitis C virus (HCV)	25% of hepatocellular carcinoma Non-Hodgkin B-cell lymphomas	America Asia North Africa South Europe
Epstein–Barr virus (EBV)	40% of Hodgkin lymphoma >95% of endemic Burkitt lymphoma 10% gastric carcinoma Nasopharyngeal carcinoma Kaposi sarcoma Other lymphomas	America East Asia East Africa
Human T-cell leukemia virus 1 (HTLV-1)	>99% of adult T-cell leukemia	Africa Australia Japan Middle East South America
Kaposi sarcoma-associated herpesvirus (KSHV/HHV-8)	>99% of Kaposi sarcoma >99% of primary effusion lymphoma	Europe Sub-Saharan Africa
Merkel cell polyomavirus (MCPyV)	80% of Merkel cell carcinoma	Australia Europe North America

## Data Availability

Not applicable.
